# How a woman's interpersonal relationships can delay care-seeking and access during the maternity period in rural Zambia: An intersection of the Social Ecological Model with the Three Delays Framework

**DOI:** 10.1016/j.socscimed.2018.11.011

**Published:** 2019-01

**Authors:** Jeanette L. Kaiser, Rachel M. Fong, Davidson H. Hamer, Godfrey Biemba, Thandiwe Ngoma, Brittany Tusing, Nancy A. Scott

**Affiliations:** a Department of Global Health, Boston University School of Public Health, 801 Massachusetts Avenue, Crosstown Center 3rd Floor, Boston, MA, 02118, USA; b Section of Infectious Diseases, Department of Medicine, Boston Medical Center, One Boston Medical Center Pl, Boston, MA, 02118, USA; c Zambia Center for Applied Health Research and Development, Plot 4186 Addis Ababa Drive, Long Acres, P.O. Box 30910, Lusaka, Zambia

**Keywords:** Zambia, Maternal health, Skilled birth attendance, Facility delivery, Interpersonal, Three delays, Social Ecological Model, Qualitative

## Abstract

To reduce maternal mortality, countries must continue to seek ways to increase access to skilled care during pregnancy and delivery. In Zambia, while antenatal attendance is high, many barriers exist that prevent women from delivering with a skilled health provider. This study explores how the individuals closest to a pregnant woman in rural Zambia can influence a woman's decision to seek and her ability to access timely maternity care. At four rural health centers, a free listing (n = 167) exercise was conducted with mothers, fathers, and community elders. Focus group discussions (FGD) (n = 135) were conducted with mothers, fathers, mothers-in-law, and community health workers (CHWs) to triangulate findings. We analyzed the FGD data against a framework that overlaid the Three Delays Framework and the Social Ecological Model. Respondents cited husbands, female relatives, and CHWs as the most important influencers during a woman's maternity period. Husbands have responsibilities to procure resources, especially baby clothes, and provide the ultimate permission for a woman to attend ANC or deliver at a facility. Female relatives escort the woman to the facility, assist during her wait, provide emotional support, assist the nurse during delivery, and care for the woman after delivery. CHWs educate the woman during pregnancy about the importance of facility delivery. No specific individual has the role of assisting with the woman's household responsibilities or identifying transport to the health facility. When husbands, female relatives, or CHWs do not fulfill their roles, this presents a barrier to a woman deciding to deliver at the health facility (Delay 1) or reaching a health facility (Delay 2). An intervention to help women better plan for acquiring the needed resources and identifying the individuals to escort her and those to perform her household responsibilities could help to reduce these barriers to accessing timely maternal care.

## Highlights

• First use of the Three Delays Framework combined with the Social Ecological Model.
• Free listing and focus group discussions conducted with communities in rural Zambia.
• Husbands, female relatives, CHWs influence a woman's access to maternity care.
• Unfulfilled roles are barriers to women seeking and delivering at a health facility.
• Birth preparedness intervention utilizing CHWs could reduce the interpersonal barriers.

## 1 Background

The Sustainable Development Goals (SDGs) set a global maternal mortality ratio (MMR) target of less than 70 deaths per 100,000 live births by 2030 (United Nations, 2017), an ambitious goal, particularly for countries such as Zambia, where the MMR was 398 per 100,000 live births in 2014 (Central Statistical Office (CSO) [Zambia], Ministry of Health (MOH) [Zambia], & ICF International, 2015). To reach Zambia's own target of reducing MMR to 162 deaths per 100,000 live births by 2021 (Ministry of Health (MOH) [Zambia], 2017), Zambia must continue to increase access to health services for antenatal, laboring, and postpartum women. One key strategy is to ensure women receive services from skilled providers throughout the maternity period as recommended by the World Health Organization (WHO) (World Health Organization, 2004, 2013, 2016). In Zambia, while 98% of pregnant women nationwide attend at least one antenatal care (ANC) visit during pregnancy, only 56% attend the recommended four or more visits (Central Statistical Office (CSO) [Zambia] et al., 2015). Only 64% of deliveries occur in the presence of a skilled birth attendant nationally, dropping to 52% in rural areas (Central Statistical Office (CSO) [Zambia] et al., 2015). Furthermore, only 63% of women nationally and 54% of rural women attend postnatal care visits within the first two days after delivery (Central Statistical Office (CSO) [Zambia] et al., 2015). While any ANC attendance is high, many barriers still exist that prevent women, especially rural women, from receiving the necessary maternity services under the care of a skilled provider.

The Three Delays Framework has been widely used to conceptualize critical points where barriers associated with accessing maternity health services can occur: 1) delay in the decision to seek care; 2) delay in arrival to a facility; and 3) delay in the provision of adequate care (Thaddeus and Maine, 1994). While this conceptual framework was first developed to understand health care decision-making and access to care for complications during a home delivery, it has been adapted to understand decision-making and access around delivery location as well (Gabrysch et al., 2009). This updated model groups factors that can result in the delays into six main categories: (1) sociocultural factors, (2) perceived benefit/need, (3) economic accessibility, (4) physical accessibility, (5) quality of preventive care, and (6) quality of emergency care. These barriers, or the perceived existence of these barriers, can influence the decision to seek preventive care for delivery, and accessibility can influence the ability to reach care.

While the Three Delays Model has been a standard and valid method of assessing barriers to care around maternity, it has been considered too simplistic or restricted in its groupings (Bohren et al., 2014; Knight et al., 2013; Sorensen et al., 2011). The Social Ecological (SEM) Model takes a broader view of multi-level factors that influence health behaviors and is useful for guiding complex interventions. Frequently depicted as a series of concentric circles, the SEM includes: (1) individual characteristics, knowledge, attitudes, and skills (Individual level) at the centre; (2) relationships with family, friends, and those within their close social networks (Interpersonal level); (3) the structural, cultural, and services environment within their “local” community (Community level); and (4) the greater social, cultural, economic, and policy structures (Societal level) within which an individual exists (McLeroy et al., 1988; Smedley and Syme, 2000). Though widely applied in health behavior research (Challa et al., 2018; Clark et al., 2017; Larios et al., 2009), we only found one study that explicitly applied the SEM to frame factors influencing maternal care-seeking behaviors (Shahabuddin et al., 2017).

When applied retrospectively to previously conducted studies, the majority of research on the influencers of maternity care-seeking behaviors examines factors associated with the Individual and Community levels of the model (Gabrysch et al., 2009; Moyer and Mustafa, 2013). Falling within the Individual level of the SEM, maternal age, education, religion, ethnicity, parity, socioeconomic status, and attitudes toward the importance of facility delivery have been widely studied as influencers of maternal care-seeking behavior (Moyer and Mustafa, 2013). Within the Community level of the SEM, urban versus rural residence, distance to health services, availability of transport, and the quality and availability of services, have also been widely studied as influencers of maternal care-seeking (Moyer and Mustafa, 2013). However, less attention has been paid to the Interpersonal level of the model, particularly how those within a woman's family and social network influence her care-seeking behavior and eventual delivery location (Moyer and Mustafa, 2013; Påfs et al., 2016). Previous studies, primarily in East and West Africa, have examined the effects of polygamy, women's empowerment/autonomy, husband's characteristics, permission from the husband or family, and the social influence of peers (Aborigo et al., 2018; Iganus et al., 2015; Kwambai et al., 2013; Moyer and Mustafa, 2013; Påfs et al., 2016; Sialubanje et al., 2015); but little has been observed in Southern Africa, particularly the Zambian context. Examination of a woman's interpersonal relationships and how those relationships can result in barriers to maternity care-seeking behaviors and access in the Zambian context is warranted.

This qualitative analysis explores how a woman's interpersonal relationships and how the roles and responsibilities of the most prominent individuals in a woman's life influence her decision to seek (Delay 1), and ability to access (Delay 2) maternal care - the intersection between the SEM and the Three Delays Framework – in rural Zambia.

## 2 Methods

### 2.1 Study design and setting

This cross-sectional study was conducted as the formative evaluation for a maternity waiting home (MWH) intervention in Zambia (Henry et al., 2017; Scott et al., 2018a, 2018b). Data were collected in the catchment areas of four rural health centres in the contiguous districts of Choma and Kalomo in Southern Province, Zambia. The districts are culturally and demographically similar, with comparable health statistics. Choma and Kalomo have primarily rural (76% and 93%, respectively) populations of approximately 250,000 (Central Statistical Office Zambia, 2014). The majority of the people are ethnically Tonga and Tonga-speaking (89% and 95%, respectively) (Central Statistical Office Zambia, 2014). Approximately 26% of married women in Southern Province are in a polygamous relationship with a husband and one or more co-wives (Central Statistical Office Zambia, 2014). Both Choma and Kalomo districts also have high total fertility rates of 6.5 and 7.3, respectively, and relatively high infant mortality rates of 59 and 66 deaths per 1000 live births (Central Statistical Office Zambia, 2014). At the time of data collection in late 2013, Choma and Kalomo districts included what are now Pemba and Zimba districts, respectively. The most recent provincial MMR for Southern Province was slightly lower than the national MMR of the same time period (343 vs. 398 deaths per 100,000 live births, respectively) (Ministry of Health (MOH) Zambia & Ministry of Community Development Mother and Child Health (MCDMCH) Zambia, 2013).

In Kalomo, the Saving Mothers, Giving Life (SMGL) initiative aimed to rapidly improve maternal health outcomes by mobilizing community members around the issue of maternal health (Kruk et al., 2014), including training a cadre of community health workers (Safe Motherhood Action Groups, SMAGs) (Jacobs et al., 2018; Sialubanje et al., 2017) responsible for improving the knowledge of and access to maternal health services within their local communities. SMGL also mentored health facility staff to increase quality of care, worked to improve the referral system, and invested in the supply chain and facility equipment (Kruk et al., 2014). Evidence suggests that SMGL had an impact on the health system and its provision of maternal health care in Kalomo district (Kruk et al., 2016; Henry et al., 2018). The data for this analysis were collected while the SMGL intervention was being implemented in Kalomo district, but before SMGL expanded to Choma district.

### 2.2 Data collection

Within the catchment areas of the four rural health facilities, we selected villages at varying distances from the health facility (<5 km, 5–10 km, >10 km), and randomly selected households within villages for a short household survey. We conducted the survey with women who were pregnant or had a child under the age of two, men with a child under the age of two, and community elders, defined as any community member over the age of 54. The survey captured the basic demographics of the respondents as well as practices and perspectives around delivery and the local MWH, and willingness to pay for MWH services (Scott et al., 2018b; Vian et al., 2017).

We then used free listing (Bernard, 2013), a qualitative approach asking participants to respond to a derivative of the same broad, open ended question in order to rapidly generate an exhaustive list of responses to understand how and what participants perceive as support to pregnant women in their communities. The following question was asked to each participant, with the prompt changing depending on the participant:

What are the important tasks that *people/men/elders* in your community do regularly to care for pregnant women through delivery and the first few days after delivery?

The intent of the question was to generate an understanding of how people who have interpersonal relationships with pregnant women perceive how they support pregnant women in their communities. The results for pregnant and recently delivered women reflect what they believe to be the most important roles for people in general in their lives, while the responses by men and elders reflect what they believe to be the most important roles of men and elders, respectively.

We also conducted focus group discussions (FGD) with (1) women who were pregnant or had a child under the age of two; (2) men with a child under two years of age; (3) mothers-in-law; and (4) traditional birth attendants (TBAs) or SMAGs (Kalomo sites only). Participants for the FGDs were recruited by health facility staff or community health workers (CHWs) and purposively selected to participate.

The FGD guides broadly assessed the pregnancy experience. For example, the first section asked: Tell me about the customs and traditions in your community for pregnant women, women giving birth, and during the few days after delivery. This was followed by a set of probes informed by the free list responses about where women deliver and who provides permission or support. Socio-demographic characteristics of all FGD respondents were collected. All data were collected in a private space and in the language most comfortable for the respondent.

### 2.3 Data analysis

Demographic data for the free list and FGDs were captured on paper forms, entered into CSPro v5.0 (United States Census Bureau), and analyzed using SAS v9.4 (SAS Institute Inc., Cary, NC). We used pile sorting (Bernard, 2013; Yeh et al., 2014) nightly to detect emerging themes from the free list responses; detailed analysis methods have been reported elsewhere (Scott et al., 2018b). The most frequently emerging themes were explored in more depth in the FGDs. Data are presented in aggregate. Elders were not stratified by gender because we did not observe any notable differences in responses.

FGDs were audio recorded, translated into English, transcribed into Microsoft^®^ Word, and analyzed in NVivo v11 (QSR International, Doncaster, Australia). We analyzed the FGD data against a framework that overlaid the Three Delays Model and the SEM, explained in more detail below. We coded each transcript to the domains of each model and conducted a content analysis in which codes were progressively grouped into larger themes (McLeroy et al., 1988) to understand the dynamics of interpersonal relationships on the first and second delays.

### 2.4 Theoretical framework

**Fig. 1:**
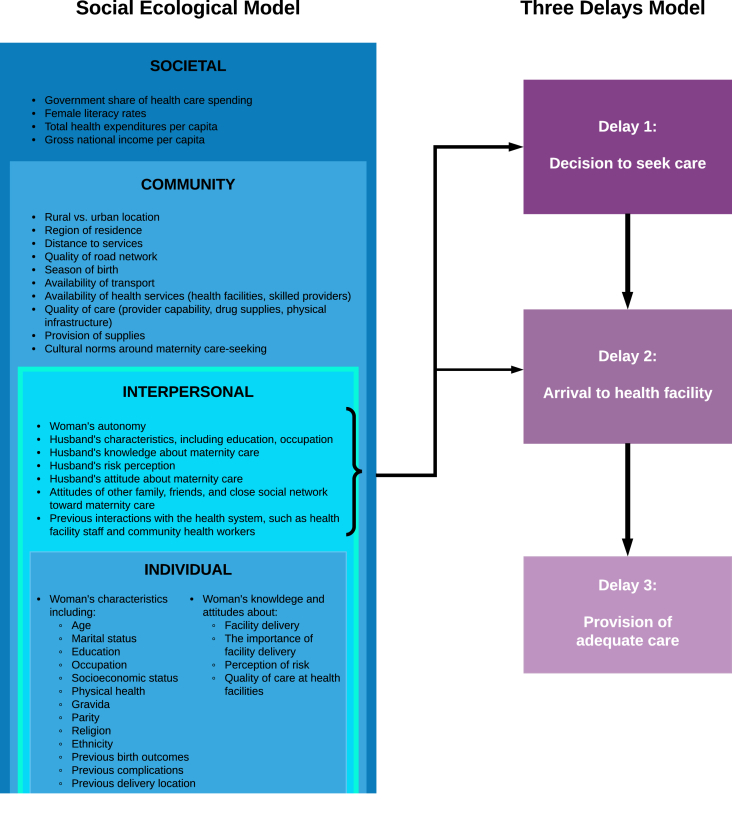
**The theoretical framework.** This figure reflects the intersection of the interpersonal level of the Social Ecological Model and the Three Delays Framework.

The Three Delays Model has been the standard framework used to conceptualize critical stages at which barriers to maternity care can occur, but it does not identify *how* barriers occur. We, therefore, combined this model with the SEM to explore the interplay of a pregnant woman's support network that may influence her seeking and reaching care, from the proximal (Individual attributes) to the distal (Societal context) factors. To guide our understanding and analysis of the FGD data, we focused on the intersection of domains in the Three Delays Model and the SEM (Fig. 1). We utilized the constructs within three systematic reviews on barriers and facilitators to facility delivery (Bohren et al., 2014; Gabrysch et al., 2009; Moyer and Mustafa, 2013) to populate the SEM and then related those influencers to the Three Delays Model (Fig. 1).

### 2.5 Ethical considerations

Ethical approval for the study was granted from the Boston University Institutional Review Board (IRB) and the ERES Converge IRB in Lusaka. We secured support for the study from the Ministry of Health and traditional leaders in the study villages. Data collectors were fluent in English and Tonga and attended a five-day training. We obtained verbal informed consent from each participant, as deemed appropriate by both IRBs. Respondents were all 18 years of age or older.

## 3 Results

### 3.1 Demographics

A total of 167 free list respondents and 135 FGD respondents from 17 FGDs participated in this study (Table 1). Approximately a third of free list respondents lived less than five, five to ten, and greater than ten kilometers from the health facility, respectively. Women free list respondents were younger than their male counterparts. Women FGD respondents were also younger than their male counterparts; both groups were younger than the mothers-in-law and TBA and SMAG groups. The majority of FGD respondents lived between five and ten kilometers from the health facility. Among the women FGD respondents, most (84.8%) delivered their last child or planned to deliver their current pregnancy at a health facility or hospital; responses from male counterparts were missing too much data to be reliably reported.

**Table 1:** Characteristics of the free list and focus group discussion respondents.

Free List Participants			
	Women (n = 59)	Men (n = 53)	Elders (n = 55)
Age, median (IQR)	25 (22,33)	32 (28,37)	63 (56,70)
Female, n (%)	59 (100)	–	26 (47)
Marital Status, n (%)			
*Married or cohabitating*	54 (91.5)	53 (100)	41 (74.6)
*Widowed or divorced*	3 (5.1)	–	14 (25.4)
*Single, never married*	2 (3.4)	–	–
Gravida, mean (SD)	3.9 (2.1)	5.6 (4.2)	–
Parity, mean (SD)	3.7 (2.0)	5.3 (3.8)	–
Delivery location for last child			
*Health facility or hospital*	37 (62.7)	40 (75.5)	–
*Home or other*	22 (37.3)	13 (24.5)	–
Distance from health facility, n (%)			
<5 km	22 (37.3)	16 (30.2)	16 (29.1)
5 – 10 km	21 (35.6)	18 (34.0)	21 (38.2)
>10 km	15 (27.12)	19 (35.8)	18 (32.7)

Focus Group Discussion Participants				
	Women (n = 33)	Men (n = 32)	Mothers-in-law (n = 32)	TBAs and SMAGs (n = 38)
Age, median (IQR)	23 (18,29)	34 (29,44)	57 (51,59)	50 (45,56)
Female, n (%)	33 (100)	–	32 (100)	31 (81.6)
Marital status, n (%)				
*Married*	30 (90.9)	31 (100)	18 (58.1)	29 (76.3)
*Widowed or divorced*	1 (3.0)	–	12 (38.7)	9 (23.7)
*Single, never married*	2 (6.1)	–	1 (3.2)	–
Distance from health facility, n (%)				
<5 km	10 (30.3)	10 (32.3)	8 (25.0)	12 (32.4)
5 – 10 km	12 (36.4)	15 (46.9)	20 (62.5)	18 (48.7)
>10 km	11 (33.3)	7 (21.9)	4 (12.5)	7 (18.9)

SD = standard deviation; IQR = interquartile range.

### 3.2 Free list results

In response to the questions about caring for pregnant women through delivery and early postpartum, women most frequently cited doing housework, being escorted to the clinic, being provided with baby clothes and nutritious food, and having her belly massaged as the most important responsibilities that people in their lives have during the maternity period (Table 2). Men highlighted the importance of providing baby clothes, doing housework, escorting the woman to the clinic for delivery, and providing nutritious food to the pregnant woman. Elders reported their most important responsibilities were to perform the woman's housework and massage her belly and back to prevent blood clots. Approximately one third of elders cited their role as escorting the woman to the clinic, providing baby clothes or nutritious food, or educating the woman on maternity topics. Fewer respondents noted that it is anyone's responsibility to assist the woman in getting transport to the clinic, to administer traditional medicine, or to care for the woman's young children.

**Table 2:** Top ten most frequently cited responsibilities of families and communities in caring for pregnant/delivering women, as reported by free list respondents, n (%).^a^

		Women n = 59	Men n = 53	Elder n = 55	Total n = 167
1	Do housework for the woman when she is nearing delivery, including light housework, fetching water, grinding maize, etc.	45 (76.3)	41 (77.4)	38 (69.1)	124 (74.3)
2	Escort woman to deliver at clinic	36 (61.0)	36 (67.9)	21 (38.2)	93 (55.7)
3	Provide baby clothes before the woman delivers	29 (49.2)	44 (83.0)	17 (30.9)	90 (53.9)
4	Provide nutritious food to the pregnant woman at home or at the MWH	29 (49.2)	33 (62.3)	20 (36.4)	82 (49.1)
5	Massage the woman's belly and back with a hot compress or give the woman hot water to encourage blood flow and prevent blood clots	26 (44.1)	6 (11.3)	37 (67.3)	69 (41.3)
6	Cook for the woman after delivery	13 (22.0)	5 (9.4)	11 (20.0)	29 (17.4)
7	Advise and educate the woman on maternity topics including ANC, delivering at a facility, not doing housework during pregnancy, and newborn care	5 (8.5)	6 (11.3)	17 (30.9)	28 (16.8)
8	Assist the woman with transport to the clinic or saving money for transport	11 (18.6)	9 (17.0)	4 (7.3)	24 (14.4)
9	Give traditional medicine to the woman and/or baby	11 (18.6)	0 (n/a)	12 (21.8)	23 (13.8)
10	Care for the woman's young children	7 (11.9)	4 (7.5)	4 (7.3)	15 (9.0)

a Remaining responses were too few within the whole sample to be included in this table.

### 3.3 Focus group discussion results

#### 3.3.1 Roles and responsibilities

Table 3 presents results stratified by roles and key emerging themes with illustrative quotes. FGD respondents consistently discussed the roles of husbands; female relatives, including the mother, mother-in-law, grandmother, aunts, or sisters; and TBAs/SMAGs. Roles ranged from providing financial support or resources, to physical support by doing household chores and escorting the woman to the clinic, and emotional support during labor.

**Table 3:** Roles and responsibilities of husbands, female elders, and TBA/SMAG toward women during pregnancy, delivery, and postpartum.

Responsibility of:	Theme	Time Period	Role	Illustrative Quotes
Husband	Provide resources for pregnancy and delivery	During pregnancy, in preparation for delivery	• Buy baby clothes • Buy requirements for facility delivery (including cord clamps, bleach, and a razor blade) • Provide nutritious food for the woman at home or for her to take to the MWH	• “The husband should buy baby clothes, baby blankets, soap. So the husband should take responsibility to buy all these before time comes for delivery. That is how he should support (her).” – FGD with men • “We also give food that will help produce milk for the baby from the mother.” – FGD with men • “A pregnant woman gets support from her husband who will work for money to feed her or even bring her to the clinic.” – FGD with TBA/SMAG
	Decision-maker on delivery location	During pregnancy, in preparation for delivery	• Head of the household • Provides permissions for the woman to go the health facility • Can refuse wife from going to the health facility	• “The husband makes health decisions and at times even his wife also can make decisions together with the husband.” – FGD with men • “Yes, my husband had to allow me to come and stay at the shelter (MWH).” – FGD with women • “The husband is supposed to support and give permission to come and use the shelter (MWH).” – FGD with mothers-in-law
Female Relative	Escort woman to health facility	Before or during delivery	• Escort woman to health facility or MWH • If delivered at home, escort woman to health facility if any complications	• “Even the mother-in-law will escort the woman to come and wait for her delivery day. Anyone of her family members can escort the woman.” – FGD with mothers-in-law • “The elders help her and sees that she has delivered well and they take her to the clinic if there is any complication.” – FGD with men
	Assist during delivery	During delivery	• Call nurse when labor starts • Assist nurse during delivery • Provide moral support • Bring mother food and water • Advise on the position in which to lie • Assist woman to push • Cut and tie cord (for home deliveries only) • Dispose of placenta (home and facility deliveries) • Receive baby from nurse	• “The caretaker will see to it when you are about to deliver she will go to call the nurse.” – FGD with women • “There are so many things the care taker can assist (with), sometimes the woman will ask to be massaged on the back due to pain and also advise on the position the mother should lie.” – FGD with men • “When she receives the baby, she is supposed to quickly cut the umbilical cord and making sure that the baby is fine.” – FGD with mothers-in-law • “The nurse and the caretaker of the mother are usually present because the nurse is the one conducting the delivery, the caretaker is to be near in case the nurse wants to be handed something, such as baby clothes, and she is the one to wash the soiled linen.” – FGD with mothers-in-law
	Assist woman immediately postpartum	Immediately or within first few hours after delivery	• Hot compress for woman • Bathe woman • Wipe and wrap baby, put on woman's chest • Clean blood and wash linens • Cook for woman	• “They also give a hot compress so that if there are blood clots, they will come out.” – FGD with TBA/SMAG • “We also clean the mother and give her a bath. We also cook food for her and (give her a) hot compress.” – FGD with mothers-in-law
	Uphold family traditions and customs	Immediately or within first few hours after delivery	• Teach traditions to the woman • Ensure the woman does not cook any food until the umbilical cord drops • Use traditional medicine to bathe the baby to protect it from illness	• “(For) three weeks after delivery the mother is not supposed to do any hard work … to avoid bleeding.” – FGD with men • “A mother should not cook anything until the cord drops. The elders say it is a big taboo, that will delay (it) to drop. (It can) take a longer time (to drop) and it can cause other problems” – FGD with men • “They put maize stem at the mother's house to show that there is a newborn baby so that pregnant women cannot visit. (If pregnant women visit,) the baby can become sick of *luhumwe* (extended abdomen).” – FGD with women • “We also prepare some traditional medicine for the baby to drink. The medicine is for protection against *luhumwe.*” – FGD with mothers-in-law • “*Nsambilo* is … a shrub-like tree. You take the roots, soak it in water and you can use it to bath a baby for at least one month so that the baby can be fat.” – FGD with women
TBA/SMAG	Educate pregnant woman	During pregnancy	• SMAGs teach community members: danger signs, nutrition, benefits of health facility delivery, and not to use traditional medicine during pregnancy • TBAs encourage community to come to wait at MWH and deliver at the health facility	• “SMAG members teach pregnant women and their husbands the need to deliver at the clinic” – FGD with mothers-in-law • “SMAG go around in the villages to teach the community not to use African medicine. Some have stopped using African medicine. We tell (a woman) that if she is not feeling well she has to run to the clinic.” – FGD with TBA/SMAG • “SMAGs and us TBAs are the ones responsible to (ensure) the safe delivery of each mother” – FGD with TBA/SMAG
Unspecified Individual/No-consensus on person responsible	Help woman with household chores	During late pregnancy and delivery	• Cook for husband • Care for children • Find transport to go to health facility	• “We support them by looking after the young children left at home. Even if you don't look after the young children at home for the woman, you can send your (older) child to go look after the woman's child.” – FGD with mothers-in-law • “Yes, family members help you to find transport and money to use while at the shelter, they also buy clothes for the baby.” – FGD with women

#### 3.3.2 The husband: provider & decision-maker

Respondents explained that the role of the husband is most important during the pregnancy period, with men having few responsibilities during or after delivery. The husband's role is to provide resources for pregnancy and delivery, and decide where the woman would deliver or grant permission once the woman has decided.

Across all FGDs, respondents agreed preparations for delivery include procuring baby clothes and delivery supplies (i.e.: cord clamps, bleach (JIK), razor blade) and obtaining nutritious food for the woman. Most participants perceived this as a husband's job, but many acknowledged that husbands frequently do not prepare so other family members must intervene. Though few respondents expressed a need to save money, they noted that the family and woman should procure the items in preparation for delivery when the husband does not, as illustrated below:

“Some the family members help buying baby clothes and blankets, maybe your husband did not buy anything” – FGD with women

“Yes, the family members support the pregnant women by providing the requirements (for delivery) if she can't afford them on her own.” – FGD with mothers-in-law

Respondents perceive these items as required by the facility and expressed concern about stigma from providers if they do not have them when they present for delivery. Respondents often mentioned they would not go to the facility to deliver without the required items because the nurse will ask them for these items; this was most heavily discussed in FGDs with mothers-in-law and to a lesser degree among women and men:

“Some they don't have baby clothes because their husbands don't buy them. Now if I go and stay at the shelter (MWH), when I deliver, the nurse will ask for baby clothes (and) what am I going to say?” – FGD with mothers-in-law

“Others (who deliver at home) it's because they don't have required items, maybe she has only old baby clothes and would not want to be rebuked by the nurses.” – FGD with women

“We cannot manage to buy baby clothes. So, we are shy to send our wives to the clinic without those new clothes. Instead we tell our wives to deliver at home.” – FGD with men

Men report that the decision to go to the health facility for delivery should be made jointly by the husband and wife, if married. Women and elders frequently reported that the husband decides on the delivery location unless the woman is single, then she decides, often with input from female relatives.

Women and elders frequently expressed the decision-making process as getting a ‘husband's permission’ to go to the facility. Women and elders reported that husbands deny permission because men: 1) want women to be home for cooking, caring for children and sexual intimacy; 2) do not want a male nurse attending to their wife; 3) have a lack of knowledge in general and specifically about safe pregnancy and delivery or about the benefits of delivering at a health facility; 4) fear being tested for HIV (primarily reported by elders); and 5) are not motivated to make it possible for the woman to deliver at the health facility as indicated by not procuring necessary requirements (primarily reported by elders and SMAGs) (Table 4).

**Table 4:** Reasons a husband denies permission for his wife to deliver at a health facility, according to FGD respondents.

Reason that husbands denied permission	Illustrative Quotes
1) Want women to be home for cooking, caring for children and sexual intimacy	“Some husbands will stop their wives from going to the clinic. They will say that there is work at home and nobody will remain with the other children if (she) goes to the clinic.” – FGD with mothers-in-law
	“Some (women) are refused by their husband because of work at home, like this time of the year people are busy in the fields so they will stay and work.” – FGD with mothers-in-law
	“Some the husbands don't want their wives to go because they want to be with them for intimacy.” – FGD with women
2) Do not want a male nurse attending to their wife's labor	“Some (women) are refused by the husbands because they don't want the male nurse to deliver their wives.” – FGD with women
	“Some husbands think that if my wife goes to the clinic and will be delivered by a male nurse. They don't like that.” – FGD with men
3) Lack knowledge	“Some husbands have little knowledge on the importance of risks and dangers of pregnancy that can arise any time.” – FGD with men
	“Some husbands are just difficult. They have no knowledge, they don't know the benefit of delivering at a health facility.” – FGD with mothers-in-law
4) Fear of being tested for HIV	“Some women deliver at home because their spouses refuse to go for HIV testing which is a requirement by health facilities and end up delivering at home.” – FGD with mothers-in-law
	“Others they fear to be tested for HIV.” – FGD with men
5) Not motivated to assist the woman, have not procured baby clothes or other requirements	“The thing is if a husband does not care what is going on with his wife, the husband is not involved in buying baby clothes and things that are wanted for the baby.” – FGD with TBA/SMAG
	“Some it's their husbands who refuse them (from going to the health facility), reason being they have not bought baby requirements.” – FGD with mothers-in-law

### 3.4 The female relative: widespread support and imparting traditions

All respondent types reported that female relatives have important roles during the antenatal, intrapartum, and postpartum periods (Table 3). The female relative is responsible for escorting the women to the health facility, assisting the health facility staff with tasks ranging from finding the nurse when the woman goes into labor outside of normal operating hours, disposing of the placenta (at home or at the health facility) and checking the baby after delivery. After delivery, female relatives bathe the woman, wrap the baby, clean the delivery area, wash the linens, cook for the mother and provide general care including massaging and using a hot compress on the woman's back or womb to prevent blood clots. In home deliveries, female relatives have the added responsibility of delivering the baby and cutting the cord, unless a TBA is present. If complications arise during home delivery, the female relative escorts the woman to a health facility.

The female relative is also responsible for teaching and upholding family traditions and customs (Table 3). This includes ensuring the woman does not cook until the umbilical cord drops since it is believed that cooking delays the cord dropping which could cause the baby to become sick with *luhumwe* (an extended abdomen). Other customs include bathing the baby in water infused with roots of a specific tree to protect it from illness and help it gain weight. These roles are not necessarily undertaken by the same individual.

### 3.5 TBAs and SMAGs: community educators

The main role of TBAs and SMAGs is to educate their communities (Table 3). Respondents sometimes spoke specifically about the role of the TBA versus that of the SMAG. Generally, FGD respondents from Choma highlighted the TBA's role as encouraging pregnant women and their husbands to utilize the MWH and deliver at the health facility, and referring them to the health facility if any complications arise during pregnancy or a home delivery. Respondents explained the role of SMAGs is much broader than that of TBAs due to their more extensive training. SMAGs were frequently described by respondents as important community educators about pregnancy, newborn danger signs, birth planning, benefits of facility delivery, and dangers of traditional medicine. FGD respondents also mentioned that TBAs and SMAGs will escort pregnant women to the health facility when relatives are not available.

### 3.6 The “family's” role

Other important responsibilities emerged but were not explicitly linked to individuals. For example, no individual was noted as responsible for household chores or caring for the woman's children. Lastly, while discussed as being an important responsibility, respondents did not articulate who is responsible for arranging transportation to the health facility.

## 4 Discussion

Our study explored the roles and responsibilities interpersonal relationships play in a pregnant woman's access to maternity care in rural Zambia. Respondents described the important roles that husbands, female relatives (mother-in-law, mother, grandmother, or other female family member), TBAs, and SMAGs play throughout the maternity period. Understanding the roles of the individuals closest to a pregnant, delivering, and postpartum woman can elucidate facilitators or barriers to seeking and accessing maternity care, ultimately informing more targeted interventions.

Consistent with previous studies, our findings suggest that Zambian men have the primary decision-making power in a relationship (Dumbaugh et al., 2014; Iganus et al., 2015; Kwambai et al., 2013; Sialubanje et al., 2015), though some of the male FGDs also discussed the importance of deciding jointly on delivery location with their wives, which has also been found previously (Påfs et al., 2016). Since it is often the husband's decision on where a woman can deliver, and respondents generally believed men lacked knowledge regarding maternity care, men continue to be an important target for interventions to improve maternal health. Previous studies have shown that while men control resources and serve as the final authority on where and when pregnant women should seek medical care, beyond that, they have no expectation of any further role during antenatal care and therefore find it unnecessary to attend antenatal clinics with their partners (Aborigo et al., 2018). There is now strong evidence that interventions to educate and engage men are associated with improved antenatal care attendance, skilled birth attendance, facility birth, postpartum care, birth and complications preparedness, and maternal nutrition (Tokhi M, 2018).

Having the necessary resources including baby clothes, delivery supplies, and nutritious food facilitates the woman's decision to seek (Delay 1) and reach (Delay 2) the health facility for delivery whereas not having these items is stigmatizing and a factor in the decision not to use a facility for delivery. The provision of these items is primarily the husband's responsibility, as in other contexts (Iganus et al., 2015; Kalisa and Malande, 2016; Kwambai et al., 2013; Sialubanje et al., 2015, 2014). Though some husbands may not be able to procure the necessary supplies (Sialubanje et al., 2015), the responses in this study imply that when a husband does not procure the items, someone else in the woman's inter-personal network attempts to close the gap. Additionally, respondents stated that when no female relative is willing to escort the pregnant woman to the health facility for delivery, she is unlikely to seek skilled maternity care (Delay 1) or to be able to access that care (Delay 2) even if she has decided to do so. Planning early in pregnancy for supplies and for escorting the woman to the facility could facilitate access to care. Interventions targeting better planning and savings for costs associated with delivery have been shown to facilitate access to safe delivery (Ekirapa-Kiracho et al., 2017; Kaufman et al., 2017; Mutebi et al., 2017; Shaikh et al., 2017) and may be appropriate in this context.

Female relatives play many roles, including teaching women traditions, escorting the woman to the health facility or MWH, providing emotional support, bringing food and water to the woman, and assisting the nurse in basic clinical tasks. After delivery, the female relative is responsible for cleaning, disposing of the placenta, bathing the woman, wrapping the baby, cooking, and massaging the woman's belly. Additionally, while both men and women recognize childcare and continued performance of household chores as necessary for facility delivery, women are more inclined to view these as a family or community responsibility. In contrast, husbands see these more strictly as a woman's responsibility. Not having a clear individual responsible for these important tasks can impede care seeking if a woman is encouraged to remain at home instead of travelling to the health facility to deliver. Identifying individuals who will be responsible for overseeing the household responsibilities while a woman is away for delivery is important and should be incorporated into birth preparedness interventions.

Surprisingly, we observed no consensus on who is responsible for arranging transport to bring a woman to the health facility for delivery. Neither men nor elder free list respondents noted arranging transport as one of their main responsibilities. Few women free list respondents discussed the responsibility despite the fact that transport is a well-documented barrier to accessing maternity care because of the high cost (Sacks et al., 2016; Sialubanje et al., 2014), limited or no availability (Lohela et al., 2012; Munguambe et al., 2016), being unable to identify transport on short notice when labor begins (De Allegri et al., 2015; Sacks et al., 2016), long distances (Gabrysch et al., 2011; Sialubanje et al., 2014), and poor road network and quality (Munguambe et al., 2016; Sialubanje et al., 2014; Stekelenburg et al., 2004). Not having an individual responsible for acquiring transport could result in unnecessary delays, fewer options and higher transport costs. While these complex relationships are worth further investigation, working with women and their closest relatives early in their pregnancy to assign responsibility and identify transport before going into labor could mitigate this barrier.

Given their close relationship with and respect from their communities, SMAGs provide an opportunity to ensure that pregnant women and their husbands receive accurate information in order to make educated decisions regarding facility delivery. While this is true of TBAs in part, SMAGs, with their additional training, were specifically discussed as important community-based sources of messaging and education. Past studies have found educational interventions using community health practitioners to be highly effective (Ensor et al., 2014; Henry et al., 2018). When SMAGs conduct their roles well, they provide the populace with important education that informs the maternity care decision-making process. Further training of SMAGs may be necessary for them to better assist women in preparing for delivery and coordinating family members to help at the health facility and at the woman's home. Furthermore, expanding the SMGL program could have beneficial results on facility delivery, as other studies have found (Henry et al., 2018; Jacobs et al., 2018).

This study has several limitations. First, there was potential for social desirability bias among respondents who might have been hesitant to share their true beliefs with the data collectors and their peers. Second, because of the qualitative nature of the study and purposive sampling approach, findings are not broadly generalizable, though they may apply to similar cultures and settings. Third, the questions used in this analysis focused on the inter-personal support networks of pregnant women. While supply-side factors are critical elements of maternal health services and likely decision making, they were are not central to this analysis. They were explored in more detail in the overall formative evaluation (Scott et al., 2018b).

## 5 Conclusions

This study demonstrates that both the first and second delays to maternity care are heavily influenced by the interpersonal relationships of a woman during the antenatal, intrapartum and postpartum periods. Clearly understanding the roles and responsibilities of family members to support pregnant women in their planning for maternity services is necessary, but not sufficient, to address potential delays in reaching and receiving skilled delivery. Strengthening community-based initiatives such as SMAGs to help pregnant women, husbands, and their broader support networks better prepare for transportation, necessary commodities, and continued management of the household during a woman's absence may be a strategy to address the first and second delay in this context.
